# Death of Dr. W. L. Atlee

**Published:** 1879-01-20

**Authors:** W. F. Barr, Geo. E. Wiley, H. M. Grant

**Affiliations:** Committee; Abingdon, Va.; Committee; Abingdon, Va.; Committee; Abingdon, Va.


### *DEATH OF DR. W. L. ATLEE.*

At a regular meeting of Abingdon Academy of Medicine, held in the office of Dr. H. M. Grant, the following preamble and resolutions on the death of Dr. WASHINGTON L. ATLEE, of Philadelphia, Pa., were reported and unanimously adopted, the death of Dr. Atlee having been announced in appropriate remarks by Dr. W. F. Barr:

Whereas, it has pleased the Great Physician of the Universe to remove from this world, the field of his usefulness, our highly-esteemed and worthy friend, Washington L. Atlee, M. D., of Philadelphia, Pa., an Honorary Fellow of the Abingdon Academy of Medicine;

1. Resolved, That it is with deep and unfeigned regret that we chronicle his death—for the world has lost one of its greatest and best medical and surgical philosophers, teachers and practitioners; the city in which he lived, one of its best citizens; the Medical Profession, one of its most faithful and laborious members; this Academy of Medicine, one of its most prominent and highly-esteemed Fellows; and his family, its noble, kind and affectionate head.

2. Resolved, That we tender the bereaved family of the deceased our sincere condolence and heartfelt sympathy in the hour of their great affliction.

3. Resolved, That a copy of the preamble and resolutions be sent to the editors of the Abingdon *Standard* and *Virginian*, the Virginia *Medical Monthly*, the *Southern Medical Record*, the Philadelphia *Medical News*, and *Medical and Surgical Reporter*, with the request that they publish the same, and also that a copy of the same be forwarded to the family of the deceased.

W. F. BARR, M. D.,
GEO. E. WILEY, M. D.
H. M. GRANT, M. D.,
*Commitee.*

Abingdon, Va., December 2, 1878.

